# Changes in postural stability after cerebrospinal fluid tap test in patients with idiopathic normal pressure hydrocephalus

**DOI:** 10.3389/fneur.2024.1361538

**Published:** 2024-05-01

**Authors:** Eunhee Park, Sanghyeon Lee, Tae-Du Jung, Ki-Su Park, Jong Taek Lee, Kyunghun Kang

**Affiliations:** ^1^Department of Rehabilitation Medicine, School of Medicine, Kyungpook National University, Daegu, Republic of Korea; ^2^School of Computer Science and Engineering, Kyungpook National University, Daegu, Republic of Korea; ^3^Department of Neurosurgery, School of Medicine, Kyungpook National University, Daegu, Republic of Korea; ^4^Department of Neurology, School of Medicine, Kyungpook National University, Daegu, Republic of Korea

**Keywords:** normal pressure hydrocephalus, postural balance, spinal puncture, center of pressure, balance (static)

## Abstract

**Introduction:**

In patients with idiopathic normal pressure hydrocephalus (iNPH), the characteristics of balance disturbance are not as well understood as those related to gait. This study examined changes in postural stability in quiet standing after the cerebrospinal fluid tap test (CSFTT) in these patients. Furthermore, the study explored the relationship between the amount of spontaneous body sway and both gait and executive function.

**Materials and methods:**

All patients diagnosed with iNPH underwent CSFTT. We evaluated their center of pressure (COP) measurements on a force plate during quiet standing, both pre- and post-CSFTT. Following the COP measurements, we calculated COP parameters using time and frequency domain analysis and assessed changes in these parameters after CSFTT. At pre-CSFTT, we assessed the Timed Up and Go (TUG) and the Frontal Assessment Battery (FAB). We investigated the relationship between COP parameters and the TUG and FAB scores at pre-CSFTT.

**Results:**

A total of 72 patients with iNPH were initially enrolled, and 56 patients who responded positively to CSFTT were finally included. Post-CSFTT, significant improvements were observed in COP parameters through time domain analysis. These included the velocity of COP (vCOP), root-mean-square of COP (rmsCOP), turn index, torque, and base of support (BOS), compared to the pre-CSFTT values (*p* < 0.05). In the frequency domain analysis of COP parameters post-CSFTT, there was a decrease in both the peak and average of power spectral density (PSD) values in both the anteroposterior (AP) and mediolateral (ML) directions below 0.5 Hz (*p* < 0.05). In addition, the TUG scores showed a positive correlation with vCOP, rmsCOP, turn index, torque, BOS, and both the peak and average PSD values in the AP and ML directions below 0.5 Hz (*p* < 0.05). The FAB scores demonstrated a negative correlation with vCOP, rmsCOP, turns index, BOS, and both peak and average PSD values in the AP direction below 0.5 Hz (*p* < 0.05).

**Conclusion:**

In patients with iNPH who responded to CSFTT, there was an improvement in spontaneous body sway during quiet standing after CSFTT. Increased spontaneous sway is associated with impaired gait and frontal lobe function. This may be linked to impaired cortico-cortical and cortico-subcortical circuits in patients with iNPH.

## 1 Introduction

Idiopathic normal pressure hydrocephalus (iNPH), with enlarged brain ventricle and normal cerebrospinal fluid (CSF) pressure, is characterized by gait and balance disturbance, cognitive impairment, and urinary incontinence. Gait and balance disturbances are often the most prominent clinical features and the first to become apparent (1, 2). Compared with healthy individuals, the gait of patients with iNPH is characterized by a broad base, short stride length, low speed, and increased variability in stride time and length (3). The CSF tap test (CSFTT) is a widely used diagnostic and therapeutic tool for improving gait disturbance (3–6). In accordance with the Japanese guideline, clinical improvement after the CSFTT increases diagnostic certainty of iNPH from possible to probable (4). In patients with iNPH, these gait characteristics are relatively better known than balance characteristics (7).

Postural stability, also referred to as balance, is the ability of the body to maintain the center of gravity (COG) within the base of support (BOS), which is the area of contact with the support surface (8, 9). Force platforms have been used to quantify the characteristics of postural stability and calculate indirect changes in spontaneous body sway, i.e., the center of pressure (COP) calculated from ground reaction force (10, 11). The COP indicates the weighted average of all forces created from the BOS and reflects the trajectory of the COG. When the limit of stability of BOS is exceeded, an individual must take a step to reestablish the BOS below the COG to prevent a fall (12). Consequently, measuring the magnitude of COP displacement over time is related to the spontaneous joint movement needed to maintain the body against gravity (13). Additionally, the power spectral density (PSD) of COP, calculated via frequency domain analysis using Fourier transformation, provided insights into the structure of COP time series. The PSD approximates the underlying oscillations in the COP and their respective amplitude (14, 15). It is a helpful tool for evaluating the effects of small and rapid movements on spontaneous body sway during quiet standing in older adults (16), patients with Parkinson’s disease (15, 17), and patients with multiple sclerosis (18).

Balance function is clinically classified into static steady-state balance, which is the ability to maintain a steady position, such as standing, and dynamic steady-state balance, which is the ability to maintain a static position with a shift in the COG, such as walking (8, 19). Healthy individuals with a good static steady-state balance are expected to perform well in dynamic steady-state balance (19, 20). Moreover, recent studies reported the relationship between dynamic steady-state balance and cognitive function, especially executive function. Poor executive function was associated with falls and a decline in gait speed in older adults (21), patients with Parkinson’s disease (22–24), and patients with traumatic brain injury (25). Additionally, Ko et al. (26) reported that impaired executive function was associated with impaired gait function and poor responsiveness of CSFTT in patients with iNPH. However, the characteristics of static steady-state balance and the relationship between static steady-state balance and both dynamic steady-state balance and executive function in patients with iNPH have yet to be elucidated.

This study aimed to quantitatively measure the changes in postural stability, focusing specifically on spontaneous body sway during quiet standing, following the CSFTT in patients with iNPH. We measured the COP and examined changes in COP parameters using time and frequency domain analyses before and after the CSFTT. Furthermore, we investigated the potential correlation between static steady-state balance function and both dynamic steady-state balance function and executive function in iNPH patients. We hypothesized that COP parameters would improve after CSFTT compared to before the test. In addition, we proposed that increasing spontaneous body sway, indicative of postural instability, may be associated with impaired gait and executive function, which in turn can affect postural control.

## 2 Materials and methods

### 2.1 Participants

This study included patients diagnosed with iNPH, using the following criteria proposed by previous diagnostic guidelines: (1) aged >40 years, (2) symptoms that have progressed insidiously over 6 months (i.e., gait disturbance with at least cognitive impairment), (3) presented with normal CSF opening pressure, (4) showed enlarged ventricles (Evans’ ratio of >0.3) and no macroscopic obstruction of CSF flow on brain magnetic resonance imaging, and (5) positive responsiveness after CSFTT (4, 27). A lumbar tap removed 30–50 mL of CSF on each INPH patient. After the CSFTT, patients were re-evaluated with the Korean-Mini Mental State Examination (K-MMSE), the iNPH Grading Scale (iNPHGS), and the Timed Up and Go Test (TUG). Gait changes were evaluated multiple times over 7 days following the tap, and changes in cognition and urination were assessed at 1 week. CSFTT response was defined using these 3 major scales (28). INPH patients who had a positive response to the CSFTT according to the Japanese guidelines for iNPH were enrolled (28). The exclusion criteria were as follows: (1) history of stroke; (2) history of heavy alcohol use; (3) history of hospitalization due to a major psychiatric disorder; (4) history of other neurologic, metabolic, neoplastic, or musculoskeletal disorder; and (5) evidence of secondary hydrocephalus after traumatic brain injury, intracerebral hemorrhage, or meningitis.

This prospective study included patients admitted to the Department of Neurology at Kyungpook National University Chilgok Hospital between September 2021 and November 2022. Written informed consent was obtained from all participants. The Institutional Review Board of Kyungpook National University Chilgok Hospital provided ethical approval (No. 2021-07-023). All experiments were performed in accordance with relevant guidelines and regulations.

### 2.2 Assessments of gait function and frontal lobe function

We evaluated a dynamic steady-state balance function as the Timed Up and Go (TUG) test at pre-CSFTT. The TUG test measures the time it takes for a participant to stand up from a seated position in a chair, walk forward 3 meters, turn, and then return to a seated position (29).

Furthermore, we evaluated executive function as a Frontal Assessment Battery (FAB) at pre-CSFTT. The score of FAB is a short cognitive and behavioral test to assess frontal lobe functions. It consists of 6 subtests: Similarities, Verbal fluency, Motor series, Conflicting instruction, Go-no-go, and Prehension behavior. Each subtest is scored from 0 (error) to 3 (correct), with a higher score indicating better executive function associated with the frontal lobe function (30).

### 2.3 COP measurement

We assessed all participants for measuring COP at pre-CSFTT and the day after the CSFTT. We measured COP using a force-measuring plate sampled at 60 Hz (Zebris FDM-S^®^, Germany) during quiet standing with eyes opened. We instructed the participants to try to stand with their bare feet as close together as possible. For 30 s, they stood quietly on the force plate, arms held comfortably on their sides. We assessed COP twice before CSFTT (pre-CSFTT) and after CSFTT within 24–48 h (post-CSFTT).

### 2.4 Data analysis

We conducted time and frequency domain analysis of COP using Python 3.7.15^1^ and Python signal processing package SciPy 1.9.1^2^ and calculated the COP parameters using analytical methods proposed by Palmieri et al. (11) and Kotolova et al. (31).

#### 2.4.1 COP parameters using time domain analysis

We calculated the velocity of COP (vCOP) by dividing the displacement of the COP trajectory by the recording time, *t*. The anteroposterior (AP) and mediolateral (ML) directions represent the AP and ML positions, respectively.


$$\mathrm{vCOP}=\frac{\sum_{n=1}^{N}\sqrt{{\left(AP_{n-1}-AP_{n}\right)}^{2}+{\left(ML_{n-1}-ML_{n}\right)}^{2}}}{t}\;\left(\mathrm{mm}/s\right)$$

We calculated the root mean square COP (rmsCOP) as the distance between the displacement of COP and mean COP position $\left(\mu_{AP},\mu_{\mathrm{ML}}\right)$ . Then, we calculated the sum of the distances and divided it by the number of frames N during the recording time.


$$\mathrm{rmsCOP}=\frac{\sum_{n=1}^{N}\sqrt{{\left(AP_{n}-\mu_{AP}\right)}^{2}+{\left(ML_{n}-\mu_{\mathrm{ML}}\right)}^{2}}}{N}\;\left(\mathrm{mm}/\mathrm{frame}\right)$$

We calculated the turn index by dividing the sum of the COP trajectory length in each direction by its standard deviation ( $\sigma_{AP},\sigma_{\mathrm{ML}})$ in that direction; the obtained value was then divided by the recording time.


$$\mathrm{Turn index}=\frac{\sum_{n=1}^{N}\sqrt{{\left(\frac{AP_{n-1}-AP_{n}\;}{\sigma_{AP}}\right)}^{2}+{\left(\frac{ML_{n-1}-ML_{n}\;}{\sigma_{\mathrm{ML}}}\right)}^{2}}}{t}\;\left(\mathrm{mm}/s\right)$$

The torque was calculated by multiplying the weight with vCOP, where $F_{G}$ indicates each patient’s weight.


$$\mathrm{Torque}=\frac{\sum_{n=1}^{N}F_{G}\cdot \sqrt{{\left(AP_{n-1}-AP_{n}\right)}^{2}+{\left(ML_{n-1}-ML_{n}\right)}^{2}}\;}{t}\;\left(\mathrm{kg}.\frac{m}{s}\right)$$

The area of BOS was calculated as the area between both feet in contact with the force plate ( ${\mathrm{cm}}^{2}$ ), as shown in Supplementary Figure S1.

#### 2.4.2 COP parameters using frequency domain analysis

**Figure 1:**
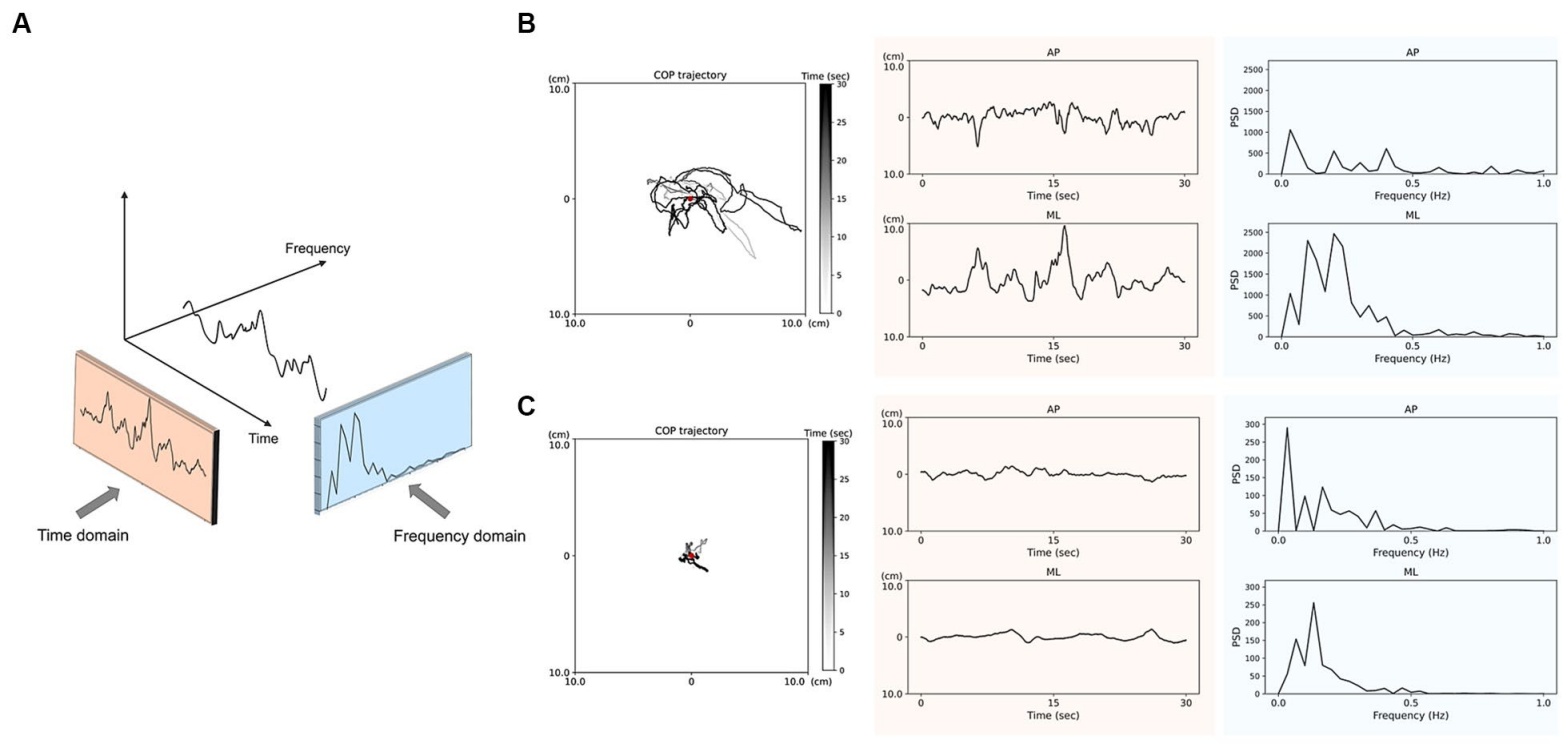
Analysis of center of pressure (COP) trajectories in the time and frequency domains **(A)** during 30-s quiet standing. The representative data of COP trajectory and displacements in anteroposterior (AP) and mediolateral (ML) directions pre-CSFTT **(B)** and post-CSFTT **(C)** for a 69-year-old male participant. The red spot indicates the center of COP displacements. The orange-colored sections represent the COP displacements over time, which is used for time domain analysis. The blue-colored sections represent the power spectral density (PSD) of COP derived from frequency domain analysis.

We quantified COP oscillations using Fourier analysis and power spectral density (PSD). Previous studies have shown that low-frequency oscillations are evident in postural sway during upright standing (14, 15, 18, 32). Additionally, considering that approximately 80% of the PSD was within 0–1 Hz range, we divided the PSD into two frequency ranges of interest: 0–0.5 Hz and 0.5–1.0 Hz. We calculated the PSD values of COP as follows: the peak PSD in AP and peak PSD in ML indicated the maximum PSD values of the AP and ML direction within 0–0.5 Hz and 0.5–1.0 Hz, and the average PSD in AP and average PSD in ML were calculated as the average value of PSD within 0–0.5 Hz and 0.5–1.0 Hz. Figure 1A illustrates the analysis of the COP trajectory in time and frequency domains during a 30-s period of quiet standing.

### 2.5 Statistical analysis

We performed all statistical analyses using SPSS software version 23 (SPSS, Inc., Armonk, NY, United States). We confirmed a normal distribution of data using the Shapiro–Wilk test (*p* < 0.05). We used a Paired *t*-test to compare changes in COP parameters using time and frequency domain analysis at pre- and post-CSFTT (*p* < 0.05, two-tailed). Furthermore, we used Pearson’s correlation to evaluate the relationship between COP parameters and both TUG score and FAB score at pre-CSFTT. We interpreted that a statistically significant correlation is shown when the correlation coefficient value (*r*) has a *p*-value of less than 0.05, indicating statistical significance. Additionally, we used Spearmen correlation to evaluate the relationship between COP parameters and subtests of FAB (Spearman correlation coefficient, rho; *p*-value < 0.05).

## 3 Results

We recruited 72 patients with iNPH, 3 of whom failed quiet standing for 30 s at pre-CSFTT, and 2 patients were lost to COP measurements after CSFTT. Also, we excluded 11 patients who did not respond to CSFTT. Finally, we included 56 patients with iNPH after positively responding to CSFTT. Of the 56 patients, 36 were male, and 20 were female (mean age 75.45 ± 5.46 years old). The average TUG score was 21.96 ± 15.39 and average FAB score was 9.78 ± 3.72 and at pre-CSFTT.

### 3.1 COP parameters using time domain analysis

The orange-colored sections in Figure 1 present representative data of the COP trajectory and displacements over time in AP and ML directions before (Figure 1B) and after (Figure 1C) CSFTT. During quiet standing at post-CSFTT, vCOP (*t* = 3.188, *p* = 0.002), rmsCOP (*t* = 2.213, *p* = 0.032), turn index (*t* = 2.483, *p* = 0.017), torque (*t* = 3.102, *p* = 0.003) and BOS (*t* = 2.550, *p* = 0.014) significantly decreased compared with those at pre-CSFTT (Table 1).

**Table 1:** Center of pressure parameters before and after CSFTT in patients with idiopathic normal pressure hydrocephalus.

COP parameters		Pre-CSFTT	Post-CSFTT	*t*	*p* value
Time domain analysis	vCOP	30.32 (12.78)	25.97 (6.95)^**^	3.188	0.002
	rmsCOP	9.49 (4.87)	8.42 (3.64)^*^	2.213	0.032
	Turns index	286.32 (384.07)	189.76 (150.27)^*^	2.483	0.017
	Torque	1.60 (0.54)	1.43 (0.37)^**^	3.102	0.003
	BOS	666.75 (141.46)	625.40 (96.67)^*^	2.550	0.014
Frequency domain analysis	Peak PSD in AP at 0–0.5 Hz	194.39 (301.61)	101.29 (107.74)^*^	2.037	0.049
	at 0.5–1.0 Hz	21.56 (27.22)	14.61 (15.21)	1.716	0.095
	Average PSD in AP at 0–0.5 Hz	63.32 (84.16)	35.86 (30.38)^*^	2.262	0.030
	at 0.5–1.0 Hz	9.10 (11.24)	5.98 (5.86)	2.026	0.050
	Peak PSD in ML at 0–0.5 Hz	472.97 (679.87)	146.06 (217.07)^**^	3.172	0.003
	at 0.5–1.0 Hz	93.29 (427.88)	11.51 (21.79)	1.236	0.224
	Average PSD in ML at 0–0.5 Hz	179.15 (320.19)	61.12 (106.06)^*^	2.289	0.028
	at 0.5–1.0 Hz	39.51 (179.85)	5.99 (14.79)	1.247	0.220

The value of mean (standard deviation). AP, anteroposterior; BOS, base of support; COP, center of pressure; CSFTT, cerebrospinal fluid tap test; rms, root-mean-square; ML, mediolateral; PSD, power spectral density; v, velocity. ^*^ represents a significant difference between pre- and post-CSFTT using paired *t*-test (^*^p < 0.05, ^**^p < 0.01).

### 3.2 COP parameters using frequency domain analysis

The blue-colored sections in Figure 1 show representative data of the PSD values in AP and ML directions before (Figure 1B) and after (Figure 1C) CSFTT. We observed a significant decrease in the peak PSD value in AP direction (*t* = 2.037, *p* = 0.049), the average PSD value in AP direction (*t* = 2.262, *p* = 0.030), the peak PSD value in ML direction (*t* = 3.172, *p* = 0.003), and the average PSD value in ML direction (*t* = 2.289, *p* = 0.028) at 0–0.5 Hz after CSFTT during quiet standing (Table 1).

### 3.3 Relationship between TUG scores and COP parameters

Table 2 is shown the relationship between TUG scores and COP parameters at pre-CSFTT in patients with iNPH. The TUG score was significantly positively correlated with vCOP (*r* = 0.523, *p* < 0.001), rmsCOP (*r* = 0.433, *p* = 0.001), turn index (*r* = 0.520, *p* < 0.001), torque (*r* = 0.421, *p* = 0.001), and BOS (*r* = 0.428, *p* = 0.001). Furthermore, TUG score was also significantly positively correlated with the peak PSD value (*r* = 0.432, *p* = 0.003) and average PSD value (*r* = 0.318, *p* = 0.033) in AP direction at 0–0.5 Hz. Additionally, TUG score was also significantly positively correlated with the peak PSD value (*r* = 0.548, *p* < 0.001) and average PSD value (*r* = 0.536, *p* < 0.001) in ML direction at 0–0.5 Hz.

**Table 2:** Correlation between clinical scores and center of pressure (COP) parameters at pre-CSFTT.

COP parameters		TUG score		FAB score	
		*r*	*p* value	*r*	*p* value
Time domain analysis	vCOP	0.523^**^	<0.001	−0.359^**^	0.007
	rmsCOP	0.433^**^	0.001	−0.270^*^	0.046
	Turns index	0.520^**^	<0.001	−0.290^*^	0.032
	Torque	0.421^**^	0.001	−0.248	0.068
	BOS	0.428^**^	0.001	−0.302^*^	0.025
Frequency domain analysis	Peak PSD in AP at 0–0.5 Hz	0.432^**^	0.003	−0.464^**^	0.002
	at 0.5–1.0 Hz	0.147	0.337	−0.171	0.266
	Average PSD in AP at 0–0.5 Hz	0.318^*^	0.033	−0.424^**^	0.004
	at 0.5–1.0 Hz	0.174	0.253	−0.247	0.107
	Peak PSD in ML at 0–0.5 Hz	0.548^**^	<0.001	−0.282	0.064
	at 0.5–1.0 Hz	0.155	0.310	−0.178	0.249
	Average PSD in ML at 0–0.5 Hz	0.546^**^	<0.001	−0.255	0.060
	at 0.5–1.0 Hz	0.125	0.414	−0.196	0.201

AP, anteroposterior; BOS, base of support; COP, center of pressure; CSFTT, cerebrospinal fluid tap test; FAB, frontal assessment battery; ML, mediolateral; TUG, timed up and go test; PSD, power spectral density; r, Pearson correlation coefficient; rms, root-mean-square; v, velocity. ^*^ represents a significant correlation between clinical scores and COP parameters using Pearson Correlation (^*^p < 0.05, ^**^p < 0.01).

### 3.4 Relationship between FAB scores and COP parameters

**Figure 2:**
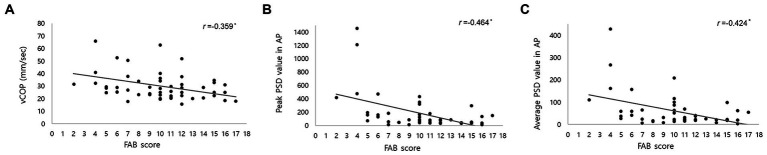
Correlation between Frontal Assessment Battery (FAB) score and center of pressure (COP) parameters, including **(A)** velocity of COP (vCOP); **(B)** the peak of power spectral density (PSD) value in anteroposterior (AP) direction below 0.5 Hz; **(C)** the average of PSD value in AP direction below 0.5 Hz. *r* represents correlation coefficient. * indicated a statistical significance in Pearson’s correlation analysis (*p* < 0.01).

We showed the relationship between FAB scores and COP parameters at pre-CSFTT in Table 2. The FAB score was significantly negatively correlated with vCOP (*r* = −0.359, *p* = 0.007), rmsCOP (*r* = −0.270, *p* = 0.046), turn index (*r* = −0.290, *p* = 0.032), and BOS (*r* = −0.302, *p* = 0.025). In addition, FAB score was also significantly negatively correlated with the peak PSD value (*r* = −0.464, *p* = 0.002) and average PSD value (*r* = −0.424, *p* = 0.004) in AP direction at 0–0.5 Hz, respectively. Figure 2 only depicts cases where the relationship between FAB scores and COP parameters is *p* < 0.01.

Furthermore, correlation between the score of subtests of FAB and COP parameters was shown in Supplementary Table S1. The score of “Similarities” was negatively correlated with vCOP (*rho* = −0.311, *p* = 0.021), peak (*rho* = −0.556, *p* < 0.001) and average PSD value (*rho* = −0.498, *p* = 0.001) in AP direction, and peak (*rho* = −0.361, *p* = 0.016) and average PSD value (*rho* = −0.355, *p* = 0.018) in ML direction at 0–0.5 Hz. The score of “Verbal fluency” was negatively correlated with vCOP (*rho* = −0.272, *p* = 0.044), rmsCOP (*rho* = −0.407, *p* = 0.002), turn index (*rho* = −0.381, *p* = 0.004), peak PSD value (*rho* = −0.346, *p* = 0.022) in AP direction, and peak (*rho* = −0.328, *p* = 0.116) and average PSD value (*rho* = −0.329, *p* = 0.029) in ML direction at 0–0.5 Hz. The score of “Motor series” was negatively correlated with vCOP (*rho* = −0.281, *p* = 0.037), torque (*rho* = −0.269, *p* = 0.047), and peak PSD value (*rho* = −0.357, *p* = 0.017) in AP direction at 0–0.5 Hz. The score of “Conflicting instructions” was negatively correlated with BOS (*rho* = −0.296, *p* = 0.028) and peak PSD value (*rho* = −0.325, *p* = 0.031) in ML direction at 0–0.5 Hz. The score of “Go-no-go” and “Prehension behavior” were not statistically significantly correlated with COP parameters.

## 4 Discussion

We investigated changes in COP parameters during quiet standing, which indicates spontaneous body sway, after CSFTT in patients with iNPH. The COP displacements associated with time domain analysis reduced after CSFTT. In addition, iNPH patients had low PSD values, indicating less variation in power value of COP in both AP and ML directions at low-frequency oscillation after CSFTT. Interestingly, impaired static steady-state balance was associated with both impaired dynamic steady-state balance and frontal lobe function.

To evaluate balance function in patients with iNPH, we measured COP during quiet standing. There have been a few studies on the quantitative measurement of balance disturbance in iNPH patients who performed shunt surgery. A previous study reported an improvement in the radius and sway area of COP after shunt surgery in 9 patients with iNPH (33). Nikaido et al. (34) demonstrated that patients with iNPH showed improved COP trajectories after shunt surgery; however, the study was limited to only 23 patients with iNPH. Furthermore, Blomsterwall et al. (35) described that patients with iNPH had a larger sway area and higher COP velocity than those with subcortical arteriosclerotic encephalopathy, but the inclusion of secondary NPH patients limited this study. To the best of our knowledge, this is the first study to investigate the characteristics of static steady-state balance function at pre-CSFTT and the changes in postural stability before and after CSFTT in patients with iNPH.

In our study, iNPH patients showed decreased COP parameters using time and frequency domain analysis after CSFTT. These changes could be interpreted as improving the ability to postural control after CSFTT. Measuring the magnitude of COP displacement over time is related to spontaneous joint movement, and calculating the PSD value helps evaluate the effect of small and rapid movements on spontaneous body sway (13, 14). Previous studies have reported significant COP displacements and higher PSD values of COP in older adults (16), patients with Parkinson’s disease (15, 17), multiple sclerosis (18), idiopathic scoliosis (36), and vestibular disorders (37) than in healthy individuals during quiet standing. Furthermore, the range of PSD is closely associated with postural control in older adults and Parkinson’s disease (14, 15). Especially, low-frequency oscillation below 0.5 Hz reflects thought to be part of the descending drive to the motor neuron pool (15, 18). The exacerbation of low-frequency oscillations probably indicates a loss of motor control of the descending drive to the motor control. This decline in motor control is likely caused by the deterioration of neurons in brain regions related to motor control (15). In this study, lower PSD values in the AP and ML direction below 0.5 Hz suggest a less frequent oscillation of spontaneous body sway during quiet standing after CSFTT. This improvement in low-frequency oscillation may be linked to an improvement in cerebral blood flow in periventricular and frontal white matter regions after CSFTT (38). Furthermore, it was suggested that motor function recovery in iNPH patients after CSF removal was related to a reversible suppression of frontal periventricular cortico-basal ganglia-thalamo-cortical circuits (39). However, the mechanisms producing balance recovery in iNPH are still not fully understood, and future studies are warranted to better investigate this aspect.

Our study found a strong correlation between static and dynamic steady-state balance at pre-CSFTT in patients with iNPH. Until now, assessments of gait function, which indicate dynamic steady-state balance, have been used as diagnostic and evaluative tools for iNPH (4, 5, 40, 41). This study suggests that measuring COP parameters during quiet standing, which assesses static steady-state balance, may be useful a potential diagnostic biomarker in iNPH patients who do not walk independently or who frequently fall. To further develop this possibility, additional studies are warranted. These should aim to quantify the differences in COP parameters between responders and non-responders of CSFTT and to compare quantitative data between static and dynamic steady-state balance, such as spatiotemporal data from gait analysis in patients with iNPH.

The spontaneous body sway was inversely associated with FAB score; in other words, poor frontal lobe functions including similarities, verbal fluency, motor series, and prehension behavior were related to more frequent oscillations of body sway. Recent studies reported the ability to balance control was related to cognitive impairment in healthy older people (42), Alzheimer’s disease (43), and Parkinson’s disease (44). Even though healthy young adults, postural control was attentionally demanding, secondary tasks could increase their spontaneous body sway (45, 46). Postural control is influenced by multifactorial brain areas related to motor control systems, including those linked to higher-level cognitive and executive functions, particularly in the frontal cortical area, as well as areas responsible for sensory feedback and coordination, such as basal ganglia, brainstem, and spinal cord (47). In patients with iNPH, ventricular enlargement may interrupt the cortical–subcortical connections that connect the frontal cortex and basal ganglia (48, 49). Furthermore, impaired uptake by glymphatic system in patients with iNPH may affect the diminished intracortical inhibitory connection between the frontal and primary motor cortices (50–53). Based on these findings, the present study revealed that impaired higher-level cognitive function in the frontal cortex areas, potentially linked to impaired cortico-cortical and cortico-subcortical circuits, is closely associated with poor balance function in patients with iNPH. These results may provide a rationale for a more thorough evaluation of postural stability and cognitive function, especially in patients with iNPH is critical understanding the disease process and exploring its potential diagnostic possibilities. Interestingly, it was reported that each subtest of the FAB might be associated with specific areas of the frontal lobes on the basis of neuropsychological, electrophysiologic, and functional arguments: conceptualization with dorsolateral areas, word generation with medial areas, and inhibitory control with orbital or medial frontal areas (30). Combining quantitative balance and neuroimaging investigations of iNPH patients may help us understand those associations and potentially any underlying pathophysiological interrelationships. Future studies are warranted to better investigate this aspect.

This study has several limitations. We measured participants’ ability to maintain a steady position during standing. Although there is a positive correlation between static and dynamic steady-state balance, it might be insufficient to explain dynamic steady-balance parameters. Recent studies have attempted to quantitatively assess dynamic characteristics during gait using a triaxial accelerometer of the trunk in patients with iNPH (54, 55). To understand postural instability in patients with iNPH, further large-scale studies are warranted to evaluate the relationship between static and dynamic steady-state balance function in patients with iNPH. Moreover, we did not compare COP parameters between patients with iNPH and healthy older controls. Healthy older adults revealed a difference in spontaneous body sway between fallers and non-fallers during quiet standing (56). Further study is needed to measure changes in COP parameters in patients with iNPH compared to older healthy adults.

## 5 Conclusion

Spontaneous body sway during quiet standing improved after CSFTT in patients with iNPH. Furthermore, the amount of spontaneous sway is associated with gait impairment and frontal lobe dysfunction. Our finding suggested that increased postural instability could be related to impaired executive functions in iNPH patients who suffered from impaired cortico-cortical and cortico-subcortical circuits.

## Data availability statement

The datasets generated and/or analysed during the current study are not publicly available because we did not get permission to disclosure it from the participants. However, it can be available from the corresponding author on reasonable request.

## Ethics statement

The studies involving humans were approved by the Institutional Review Board of Kyungpook National University Chilgok Hospital provided ethical approval (No. 2021-07-023). The studies were conducted in accordance with the local legislation and institutional requirements. The participants provided their written informed consent to participate in this study.

## Author contributions

EP: Data curation, Writing – original draft, Writing – review & editing, Formal analysis, Funding acquisition, Methodology. SL: Formal analysis, Methodology, Software, Writing – original draft, Visualization, Writing – review & editing. T-DJ: Conceptualization, Supervision, Writing – review & editing, Validation. K-SP: Conceptualization, Writing – review & editing, Supervision, Validation, Writing – original draft. JL: Formal analysis, Methodology, Supervision, Writing – review & editing, Validation, Visualization. KK: Conceptualization, Data curation, Supervision, Writing – review & editing, Validation, Writing – original draft.

## Footnotes

^1^https://www.python.org

^2^https://scipy.org
