# Supplementary material for: Changes in postural stability after cerebrospinal fluid tap test in patients with idiopathic normal pressure hydrocephalus
Source: Front Neurol. 2024 May 1;15:1361538. doi: 10.3389/fneur.2024.1361538 (PMC11094259; doi:10.3389/fneur.2024.1361538)
Supplement: Supplementary file 1 [file Table_1.DOCX]

**Table 1.** Center of Pressure Parameters Before and After CSFTT in Patients with Idiopathic Normal Pressure Hydrocephalus

| COP parameters | | Pre-CSFTT | Post-CSFTT | *t* | *p* value |
| --- | --- | --- | --- | --- | --- |
| Time Domain Analysis | vCOP | 30.32 (12.78) | 25.97 (6.95)^**^ | 3.188 | 0.002 |
|  | rmsCOP | 9.49 (4.87) | 8.42 (3.64)^*^ | 2.213 | 0.032 |
|  | Turns index | 286.32 (384.07) | 189.76 (150.27)^*^ | 2.483 | 0.017 |
|  | Torque | 1.60 (0.54) | 1.43 (0.37)^**^ | 3.102 | 0.003 |
|  | BOS | 666.75 (141.46) | 625.40 (96.67)^*^ | 2.550 | 0.014 |
| Frequency Domain Analysis | Peak PSD in AP  at 0 - 0.5 Hz | 194.39 (301.61) | 101.29 (107.74)^*^ | 2.037 | 0.049 |
|  | at 0.5 - 1.0 Hz | 21.56 (27.22) | 14.61 (15.21) | 1.716 | 0.095 |
|  | Average PSD in AP  at 0 - 0.5 Hz | 63.32 (84.16) | 35.86 (30.38)^*^ | 2.262 | 0.030 |
|  | at 0.5 - 1.0 Hz | 9.10 (11.24) | 5.98 (5.86) | 2.026 | 0.050 |
|  | Peak PSD in ML  at 0 - 0.5 Hz | 472.97 (679.87) | 146.06 (217.07)^**^ | 3.172 | 0.003 |
|  | at 0.5 - 1.0 Hz | 93.29 (427.88) | 11.51 (21.79) | 1.236 | 0.224 |
|  | Average PSD in ML  at 0 - 0.5 Hz | 179.15 (320.19) | 61.12 (106.06)^*^ | 2.289 | 0.028 |
|  | at 0.5 - 1.0 Hz | 39.51 (179.85) | 5.99 (14.79) | 1.247 | 0.220 |

The value of mean (standard deviation). AP: anteroposterior, BOS: base of support, COP: center of pressure, CSFTT: cerebrospinal fluid tap test, rms: root-mean-square, ML: mediolateral, PSD: power spectral density, v: velocity. ^*^ represents a significant difference between pre-and post-CSFTT using Paired t-test (^*^*p* < 0.05, ^**^ *p* < 0.01).

**Table 2.** Correlation between Clinical Scores and Center of Pressure (COP) Parameters at pre-CSFTT

| COP parameters | | TUG score | | | FAB score | |
| --- | --- | --- | --- | --- | --- | --- |
|  |  | *r* | | *p* value | *r* | *p* value |
| Time Domain Analysis | vCOP | | 0.523^**^ | <0.001 | -0.359^**^ | 0.007 |
|  | rmsCOP | | 0.433^**^ | 0.001 | -0.270^*^ | 0.046 |
|  | Turns index | | 0.520^**^ | <0.001 | -0.290^*^ | 0.032 |
|  | Torque | | 0.421^**^ | 0.001 | -0.248 | 0.068 |
|  | BOS | | 0.428^**^ | 0.001 | -0.302^*^ | 0.025 |
| Frequency Domain Analysis | Peak PSD in AP  at 0 - 0.5 Hz | | 0.432^**^ | 0.003 | -0.464^**^ | 0.002 |
|  | at 0.5 - 1.0 Hz | | 0.147 | 0.337 | -0.171 | 0.266 |
|  | Average PSD in AP  at 0 - 0.5 Hz | | 0.318^*^ | 0.033 | -0.424^**^ | 0.004 |
|  | at 0.5 - 1.0 Hz | | 0.174 | 0.253 | -0.247 | 0.107 |
|  | Peak PSD in ML  at 0 - 0.5 Hz | | 0.548^**^ | <0.001 | -0.282 | 0.064 |
|  | at 0.5 - 1.0 Hz | | 0.155 | 0.310 | -0.178 | 0.249 |
|  | Average PSD in ML  at 0 - 0.5 Hz | | 0.546^**^ | <0.001 | -0.255 | 0.060 |
|  | at 0.5 - 1.0 Hz | | 0.125 | 0.414 | -0.196 | 0.201 |

AP: anteroposterior, BOS: base of support, COP: center of pressure, CSFTT: cerebrospinal fluid tap test, FAB: frontal assessment battery, ML: mediolateral, TUG: timed up and go test, PSD: power spectral density, *r*: Pearson correlation coefficient, rms: root-mean-square, v: velocity. ^*^ represents a significant correlation between clinical scores and COP parameters using Pearson Correlation (^*^*p* < 0.05, ^**^ *p* < 0.01).
