# Supplementary figures and images for: Changes in postural stability after cerebrospinal fluid tap test in patients with idiopathic normal pressure hydrocephalus
Source: Front Neurol. 2024 May 1;15:1361538. doi: 10.3389/fneur.2024.1361538 (PMC11094259; doi:10.3389/fneur.2024.1361538)

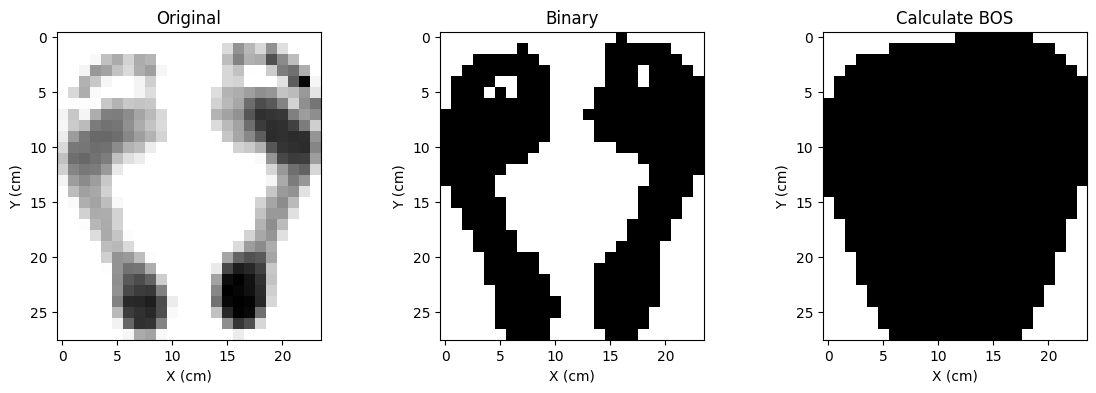

Supplement: Supplementary file 2 [file Image_1.TIF]
